# Comparison of different methods of intracerebral administration of radioiododeoxyuridine for glioma therapy using a rat model

**DOI:** 10.1054/bjoc.1999.0879

**Published:** 1999-12-08

**Authors:** R J Mairs, C L Wideman, W J Angerson, T L Whateley, M S Reza, J R Reeves, L M Robertson, A Neshasteh-Riz, R Rampling, J Owens, D Allan, D I Graham

**Affiliations:** Department of Radiation Oncology, University of Glasgow, CRC Beatson Laboratories, Garscube Estate, Glasgow, G61 1BD, UK; Department of Child Health, University of Glasgow, Royal Hospital for Sick Children, Yorkhill NHS Trust, Glasgow, G3 8SJ, UK; Department of Pathology and Neuroscience, Albert Einstein College of Medicine, Kennedy Center Room 617, 1410 Pelham Parkway South, Bronx, NY 10461, USA; Department of Surgery, University of Glasgow, Royal Infirmary, Glasgow, G31 2ER, UK; Department of Pharmaceutical Sciences, University of Strathclyde, Glasgow, G1 1XW, UK; Department of Medicine, University of Glasgow, Royal Infirmary, Glasgow, G31 2ER, UK; Radionuclide Dispensary, Western Infirmary, Glasgow, G11 6NT, UK; Department of Neuropathology, Institute of Neurological Sciences, Southern General Hospital NHS Trust, Glasgow, G51 4TF, UK

**Keywords:** radioiododeoxyuridine, glioma, intracerebral therapy, rat, sustained release

## Abstract

The Auger electron emitting agent 5-[^125^I]iodo-2′-deoxyuridine (i.e.[^125^I]IUdR) holds promise for the treatment of residual glioma after surgery because this thymidine analogue kills only proliferating cells. However, malignant cells which are not synthesizing DNA during exposure to the radiopharmaceutical will be spared. To determine whether tumour incorporation of [^125^I]IUdR could be enhanced by protracted administration, we used a C6 cell line, growing in the brains of Wistar rats, as a glioma model and compared three methods of intracerebral delivery of [^125^I]IUdR. Twenty-four hours after administration of drug, autoradiography of brain sections demonstrated nuclear uptake of the radiopharmaceutical in cells throughout tumour while normal brain cells remained free of radioactivity. The [^125^I]IUdR labelling indices (% ± s.e.m.) achieved were 6.2 (0.4) by single injection, 22.5 (4.1) using a sustained release polymer implant (poly(lactide-co-glycolide)) and 34.3 (2.0) by mini-osmotic pump. These results emphasize the need for a sustained delivery system as a prerequisite for effective treatment. These findings are also encouraging for the development of a sustained release system for radiolabelled IUdR for use in the treatment of intracranial tumours, particularly in the immediate postoperative setting. © 2000 Cancer Research Campaign


*British Journal of Cancer* (2000) **82**(1), 74–80
© 2000 Cancer Research Campaign
Article no. bjoc.1999.0879

### Comparison of different methods of intracerebral administration of radioiododeoxyuridine for glioma therapy using a rat model

**RJ Mairs[1,2], CL Wideman[3], WJ Angerson[4], TL Whateley[5], MS Reza[5], JR Reeves[4], LM Robertson[6], A Neshasteh-Riz[1], R Rampling[1], J Owens[7], D Allan[8] and DI Graham[8]**

[1]Department of Radiation Oncology, University of Glasgow, CRC Beatson Laboratories, Garscube Estate, Glasgow G61 1BD, UK; [2]Department of Child Health, University of Glasgow, Royal Hospital for Sick Children, Yorkhill NHS Trust, Glasgow G3 8SJ, UK; [3]Department of Pathology and Neuroscience, Albert Einstein College of Medicine, Kennedy Center Room 617, 1410 Pelham Parkway South, Bronx, NY 10461, USA; [4]Department of Surgery, University of Glasgow, Royal Infirmary, Glasgow G31 2ER, UK; [5]Department of Pharmaceutical Sciences, University of Strathclyde, Glasgow G1 1XW, UK; [6]Department of Medicine, University of Glasgow, Royal Infirmary, Glasgow G31 2ER, UK; [7]Radionuclide Dispensary, Western Infirmary, Glasgow G11 6NT, UK; [8]Department of Neuropathology, Institute of Neurological Sciences, Southern General Hospital NHS Trust, Glasgow G51 4TF, UK

**Summary** The Auger electron emitting agent 5-[$^{125}$I]iodo-2′-deoxyuridine (i.e. [$^{125}$I]IUdR) holds promise for the treatment of residual glioma after surgery because this thymidine analogue kills only proliferating cells. However, malignant cells which are not synthesizing DNA during exposure to the radiopharmaceutical will be spared. To determine whether tumour incorporation of [$^{125}$I]IUdR could be enhanced by protracted administration, we used a C6 cell line, growing in the brains of Wistar rats, as a glioma model and compared three methods of intracerebral delivery of [$^{125}$I]IUdR. Twenty-four hours after administration of drug, autoradiography of brain sections demonstrated nuclear uptake of the radiopharmaceutical in cells throughout tumour while normal brain cells remained free of radioactivity. The [$^{125}$I]IUdR labelling indices (% ± s.e.m.) achieved were 6.2 (0.4) by single injection, 22.5 (4.1) using a sustained release polymer implant (poly(lactide-co-glycolide)) and 34.3 (2.0) by mini-osmotic pump. These results emphasize the need for a sustained delivery system as a prerequisite for effective treatment. These findings are also encouraging for the development of a sustained release system for radiolabelled IUdR for use in the treatment of intracranial tumours, particularly in the immediate postoperative setting. © 2000 Cancer Research Campaign

**Keywords:** radioiododeoxyuridine; glioma; intracerebral therapy; rat; sustained release

Malignant glioma is the commonest primary neoplasm of the brain and has a very poor prognosis. Although it rarely metastasises, widespread local infiltration precludes complete surgical removal. Following decompression, regrowth is rapid (Vertosick et al, 1994), suggesting that the remaining malignant tissue might be particularly susceptible to cycle-specific drugs.

The Auger electron emission from $^{125}$I have an effective range of only a few nanometers (Martin and Haseltine, 1981). Therefore this radionuclide must be associated with DNA to kill cells (Kassis et al, 1987). A suitable targeting vehicle is the thymidine analogue 5-[$^{125}$I]iodo-2′-deoxyuridine (i.e. [$^{125}$I]IUdR) which is incorporated into the DNA of cycling cells. Because of their rapid rate of growth, there should be preferential uptake into tumour cells. The efficacy of locoregional administration of this agent has been demonstrated in rodent models of gliosarcoma (Kassis et al, 1998), meningeal carcinoma (Kassis and Adelstein, 1996) and ovarian ascites (Baranowska-Kortylewicz et al, 1991). Because [$^{125}$I]IUdR deiodinates rapidly in vivo (Klecker et al, 1985), it is anticipated that non-intravenous routes of administration will be employed in the initial clinical use of this agent.

Since nuclear incorporation of [$^{125}$I]IUdR occurs only in cells which are synthesizing DNA, a major limitation to this therapy is the presence within the targeted tumour of cells which are temporarily out of S phase during exposure to the drug (O'Donoghue and Wheldon, 1996). One way to overcome this restriction is to employ, in addition, radiohalogen conjugates of deoxyuridine, such as the long range β-emitter $^{131}$I (Neshasteh-Riz et al, 1998) or the α-emitter $^{211}$At (Larsen et al, 1997), whose decay particles are lethal over ranges equivalent to several cell diameters. By this approach, untargeted cells adjacent to those which have accumulated radiopharmaceutical receive a radiation dose by cross-fire. The potential benefit of this radiological bystander effect has been demonstrated using multicellular tumour spheroids (Cunningham et al, 1998; Neshasteh-Riz et al, 1998).

Alternative means of enhancing [$^{125}$I]IUdR therapy of brain tumours have also been illustrated by experimental targeted radiotherapy (Neshasteh-Riz et al, 1997). Using a spheroid model, they showed that by lengthening the time of incubation with [$^{125}$I]IUdR the cellular labelling index was increased due to the exposure of larger numbers of cells passing through S phase. Microencapsulation allows the preparation of biodegradable microspheres that can be precisely implanted in a small area of the brain by stereotactic techniques (Whateley et al, 1995; Menei et al, 1996; Reza and Whateley, 1998). IUdR can also be incorporated

*Received 11 December 1998*
*Revised 25 June 1999*
*Accepted 30 June 1999*

*Correspondence to:* RJ Mairs, Department of Radiation Oncology, University of Glasgow, CRC Beatson Laboratories, Garscube Estate, Glasgow G61 1BD, UK

into thin films of biodegradable polymer (Reza and Whateley, 1999). Recently, the efficacy of continuous infusion of [$^{125}$I]IUdR by means of micro-osmotic pump was demonstrated using an in vivo model system (Sahu et al, 1997). These findings suggest that significant sparing of non-cycling malignant glioma cells would result from treatment delivered as a single injection of [$^{125}$I]IUdR and that uptake of drug would be enhanced by prolonged exposure. We have undertaken an investigation to test this hypothesis in vivo. [$^{125}$I]IUdR was administered directly into tumours produced by intracerebral implantation in Wistar rats of C6 rat glioma cells. Three different means of delivery were employed: single injection, slow release from biodegradable inserts and diffusion from implanted osmotic-pumps. We demonstrate that the tumour cell uptake was greatly improved by prolonged delivery of the radiopharmaceutical and that the most efficient method was administration by osmotic-pump. Further development of biodegradable implantation is required to optimize this approach.

### MATERIALS AND METHODS

### Materials

Tissue culture media and supplements were purchased from Gibco-BRL (Paisley, UK). All other reagents were obtained from Sigma-Aldrich Co Ltd (Dorset, UK), unless otherwise stated. No-carrier-added [$^{125}$I]IUdR of specific activity 74 TBq mmol$^{-1}$ was obtained from Amersham International plc, UK.

### Cell culture

The C6 rat glioma cell line was obtained from the American Type Culture Collection (Rockville, MD, USA). Cells were grown in F10-DMEM (Dulbecco's modified Eagle's medium) supplemented with 10% (v/v) fetal bovine serum. The cells were passaged after reaching 90% confluency (every fourth day) and plated at an initial density of $10^4$ cells cm$^{-2}$ at 37°C in 5% carbon dioxide at 90% relative humidity. Cultures were routinely tested and found to be free from mycoplasma contamination.

### Experimental animals

All animal work was carried out in accordance with the UK Coordinating Committee for Cancer Research guidelines on experimental neoplasia in animals under the authority of a project licence granted by the UK Home Office under the Animals (Scientific Procedures) Act, 1986. Wistar male and female rats weighing 250–300 g were obtained from Harlan Olac (Bicester, UK).

### Intracranial implantation of C6 cells

The implantation procedure was similar to those described previously (Kassis and Adelstein, 1996; Zhu et al, 1996). Briefly, after the induction of anaesthesia by halothane inhalation, animals were placed in a Kopf stereotaxic frame (Clarke Electromedical, Reading, UK); the head was shaved and disinfected; a midline incision was made using a scalpel blade and underlying tissue removed using blunt dissection. Using a dentist drill fitted with a size 018 burr (Wright Dental Group, Glasgow, UK) a hole was drilled 4 mm posterior to the coronal suture and 3 mm to the right of the sagittal suture. The needle was advanced to a depth of 6 mm from the top of the skull and then withdrawn to a depth of 4 mm.

Exponentially growing C6 cells were detached from tissue culture plates using 0.1% trypsin in phosphate-buffered saline (PBS)–EDTA, suspended in PBS and counted in a Coulter counter (Coulter Electronics, Luton, UK). Using a Hamilton syringe (Hamilton, Bonaduz, Switzerland) fitted with a 23G needle, $5 \times 10^4$ cells contained in a 10 μl volume were injected slowly into the cavity. After 1 min the needle was removed.

A piece of Surgicel (Johnson and Johnson, Livingston, UK) was placed over the injection site to aid healing and prevent infection. The head wound was then closed with Dexon sutures (Davis & Geck, Gosport, Hants, UK). The procedure was carried out under sterile conditions.

The animals were observed for three weeks. Those showing signs of ill health or distress were immediately sacrificed.

### Immunohistochemistry

The choice of time after implantation of C6 cells for assessment of IUdR uptake was influenced by (a) the need to ensure a sufficient interval for growth of an evaluable tumour mass; (b) the extent of inflammatory cellular contamination; (c) the amount of necrotic tissue, and (d) the toxic effect of excessive tumour growth. To determine the time dependence of infiltration of inflammatory cells into brain lesions, animals were sacrificed at various times between 4 and 21 days after intracranial implantation of C6 cells. Rat brains and control tissues (rat thymus and spleen) were fixed in neutral buffered 4% formaldehyde for 24 h. The protocol for each antibody was optimized in control tissues prior to labelling the rat brains for the detection of T-cells, monocytes, macrophages and B-cells.

#### *T-cells*

Sections were cut at 6 μm, mounted on to silane-coated slides and baked overnight at 37°C. Sections were dewaxed and rehydrated through standard solutions and endogenous peroxidase was blocked with 3% hydrogen peroxide ($H_2O_2$) in methanol for 10 min. Antigen retrieval was carried out by boiling the sections in 10-mM citrate buffer pH 6.0 in a microwave oven for 10 min. After cooling, sections were washed in PBS (10 mM sodium phosphate, 140 mM sodium chloride, pH 7.4); non-specific binding was blocked with 20% normal goat serum for 10 minutes before the primary antibody was applied (Rabbit anti-human T-cells CD3; Dako Ltd, High Wycombe, UK) diluted to 1:400 in 10% normal goat serum and incubated overnight at 4°C. Sections were washed through 3 changes of PBS prior to application of the secondary antibody (biotinylated goat anti-rabbit IgG diluted to 1:300 in 10% normal goat serum, Dako Ltd) for 30 min. After 3 further washes in PBS, the final layer was applied: avidin–biotin–peroxidase complex (Vector Laboratories, Reading, UK). Sections were developed in 0.05% diaminobenzidine-tetrahydrochloride, 0.01% $H_2O_2$ for 10 min and labelling enhanced with 0.5% $CuSO_4$. Sections were lightly counterstained with haematoxylin, dehydrated through standard solutions and mounted in DPX.

#### *Monocytes/macrophages*

The procedure was similar to that described for the T-cells with a few exceptions. Antigen retrieval was optimized with a 10-min application of trypsin at 37°C (0.1% trypsin, 0.1% calcium chloride in distilled water). Blocking serum was 2% bovine serum

© 2000 Cancer Research Campaign albumin and the primary antibody was mouse anti-rat monocytes/macrophages (ED1, Serotec, Oxford, UK) used at a 1:400 dilution with overnight incubation at 4°C. Biotinylated rabbit anti-mouse immunoglobulins preabsorbed against rat immunoglobulins (Dako Ltd) formed the second layer and the avidin–biotin–peroxidase complex was applied as described for the T-cells.

*B-cells*

The procedure for labelling rat B-cells was essentially similar to the monocyte/macrophage protocol except that microwave antigen retrieval was performed for a total of 20 min. The primary antibody, CD79a, was mouse anti-human B-cells (DAKO Ltd). This was used at a dilution of 1:20 with overnight incubation at 4°C.

*Determination of inflammatory cell infiltrate*

Two independent observers evaluated T cell-, monocytes/macrophage- and B-cell-specific staining in 4 central and 4 peripheral fields of 6 sections (10-µm thick) from individual rat brains.

### Biodistribution of [$^{125}$I]IUdR

These experiments were performed on groups of six rats. To minimize thyroidal uptake of free radioiodide, the drinking water was supplemented with 0.1% (w/v) KI 2 days before administration of [$^{125}$I]IUdR.

One week after initiation of tumour growth and using the same coordinates as those for implantation of C6 cells, rats were injected intracranially with 0.37 MBq of [$^{125}$I]IUdR in 10 µl. The injection time was 1 min. After injection, the Hamilton syringe needle was left in place for a further minute before being slowly withdrawn. This procedure resulted in no leakage of injectate.

At various times (0.5, 1, 2, 24 and 48 h) after injection of radiopharmaceutical, the rats were killed. Tissues of interest were removed, washed with PBS, blot-dried, weighed and the radioactivity measured in an automated gamma counter (Canberra Packard, Berkshire, UK). DNA was also isolated from these tissues by phenol–chloroform extraction and the incorporated radioactivity was determined by gamma counting. Statistical analysis of biodistribution data was by Student's *t*-test.

### [$^{125}$I]IUdR-loaded biodegradable thin films

Poly(D,L-lactic-co-glycolic acid) (PLGA), 85:15 (low intrinsic viscosity, Mw 62 000), was purchased from Medisorb (Cincinnati, OH, USA). PLGA was chosen for this work because of its clinical acceptability and its biodegradable and biocompatible properties. Carboplatin and etoposide have been incorporated into PLGA implants and microspheres following studies which showed their efficacy against the C6 glioma cell line (Whateley et al, 1995).

Thin films of [$^{125}$I]IUdR were prepared by dissolving 10–30 mg PLGA in dichloromethane, mixing in small volumes (5–20 µl) of [$^{125}$I]IUdR by sonication and casting into a small siliconized petri-dish. The dichloromethane was allowed to evaporate overnight and then dried at 40°C in a vacuum oven for 24 h. Radiolabelled IUdR within the thin films has been shown to be protected from degradation during incubation in PBS, serum and plasma (Reza and Whateley, 1999).

### Implantation of [$^{125}$I]IUdR-loaded PLGA polymer

Thin films of PLGA for intracranial implantation had the dimensions 2.5 × 1.5 × 0.19 mm. These were loaded with 0.37 MBq of [$^{125}$I]IUdR. Using a dentist drill, the existing hole in the skull of a tumour-bearing rat was enlarged to a diameter of 2.5 mm. The drilling was performed in short bursts and the skull cooled with saline to prevent heat damage to the underlying brain. A fine scalpel (No. 11) was used to make an incision in the dura at a depth of 4 mm. The thin film was then gently lowered into this space. Surgicel was then placed over the craniectomy site to protect the exposed brain, prevent infection and aid healing. The wound was then closed using Dexon sutures.

### Implantation of osmotic pump

Alzet miniosmotic pumps (Charles River Ltd, Kent, UK) with a capacity of 200 µl and a flow rate of 0.14 µl per min, were filled with 200 µl of a solution containing 0.37 MBq of [$^{125}$I]IUdR in sterile saline, taking care to avoid the entrapment of air bubbles. A brain infusion cannula was then attached to the pump and the whole assembly incubated in sterile saline overnight at 37°C.

The scalp was washed, disinfected with chlorhexidine and a midline incision was made. The underlying tissue was cleared around the existing burr hole. A subcutaneous tunnel, leading from the scalp incision to the midscapular space, was made using blunt dissecting scissors. The pump was located in this space, with the catheter tubing pointing towards the skull. A plastic spacer was attached to the cannula before it was inserted into the burr hole to achieve a depth of 4.5 mm. The cannula was attached to the skull using Histoacryl (Cyanamid, Hampshire, UK). This was allowed to harden for a few minutes before the scalp was closed with Dexon sutures. This procedure was carried out under sterile conditions.

### Intracerebral distribution of [$^{125}$I]IUdR

Twenty-four hours after the initiation of the administration of 0.37 MBq [$^{125}$I]IUdR by intracranial single injection, polymer implantation or osmotic pump delivery, animals were sacrificed. Brains were prepared for sectioning and autoradiography by freezing in isopentane (BDH, Poole, UK) cooled to –42°C for 10 min and then covering in Lipshaw embedding medium. They were then stored at –20°C until required. After sectioning (10-µm thick) and mounting, the slides were dipped in a 1:1 dilution of Kodak NTB-2 emulsion in distilled water at 43°C. After drying, the sections were exposed in light-proof desiccating boxes for 92 h. Sections were developed in 1:1 dilution of Kodak D 19 developer at 10°C for 4 min. After a brief wash in distilled water, the emulsion was fixed in Kodafix for 5 min. The slides were washed and couterstained with haematoxylin, dehydrated through graded ethanols and mounted in DPX synthetic resin mountant (BDH, Poole, UK).

To determine the depth within the tumour where maximal uptake of [$^{125}$I]IUdR had taken place, the tissue of every third section was solubilized with 1 M sodium hydroxide and radioactivity was measured in a gamma counter. For each experimental animal, four sections, adjacent to the depth associated with peak activity, were selected for the estimation of IUdR labelling indices. Sections were examined using a Leitz microscope with a 10 × 10 square grid eyepiece graticule at 400× magnification, providing a

 © 2000 Cancer Research Campaign

**Table 1** Biodistribution data for a single intracranial injection of 0.37 MBq [$^{125}$I]IUdR into normal (upper values) and tumour-bearing (lower values) rats. Results are expressed as percent of injected dose per gram of tissue (mean ± s.d.); $n$ = 6. s.d. values less than 0.005 were omitted

| Time (h)<br>Organ | 0.5 | 1.0 | 2.0 | 24.0 | 48.0 |
|---|---|---|---|---|---|
| Brain | 12.7±0.82 | 10.6±0.51 | 1.9±0.30 | 0.14±0.02$^{c}$ | 0.13±0.02$^{c}$ |
| | 11.6±1.47 | 11.0±0.45 | 2.2±0.42 | 0.9±0.18 | 1.1±0.20 |
| Blood | 0.70±0.12 | 0.52±0.08 | 0.34±0.04 | 0.02±0.01 | 0.01 |
| | 0.66±0.08 | 0.46±0.06 | 0.38±0.05 | 0.02±0.01 | 0.01 |
| Thyroid | 0.56±0.09 | 0.69±0.15 | 0.47±0.05$^{c}$ | 0.44±0.06$^{c}$ | 0.46±0.05 |
| | 0.61±0.10 | 0.58±0.11 | 0.66±0.07 | 0.70±0.05 | 0.42±0.04 |
| Liver | 0.49±0.07 | 0.28±0.05 | 0.19±0.04 | 0.02±0.01 | 0.01 |
| | 0.53±0.09 | 0.33±0.07 | 0.22±0.04 | 0.02±0.01 | 0.01 |
| Small intestine | 0.97±0.18 | 0.59±0.14 | 0.39±0.07$^{b}$ | 0.20±0.05 | 0.07±0.02 |
| | 1.12±0.23 | 0.67±0.10 | 0.30±0.06 | 0.24±0.05 | 0.08±0.02 |
| Kidney | 0.41±0.03 | 0.44±0.07 | 0.33±0.06 | 0.02±0.01 | 0.02±0.01 |
| | 0.48±0.11 | 0.40±0.02 | 0.29±0.04 | 0.01 | 0.01 |
| Stomach | 1.83±0.22 | 2.07±0.38 | 1.05±0.08 | 0.37±0.09 | 0.12±0.02$^{a}$ |
| | 1.84±0.25 | 1.94±0.19 | 1.10±0.28 | 0.29±0.04 | 0.10±0.02 |
| Bone marrow | 0.20±0.03 | 0.19±0.03 | 0.16±0.02 | 0.03±0.01 | 0.03±0.01 |
| | 0.22±0.04 | 0.17±0.02 | 0.18±0.03 | 0.03±0.01 | 0.03 |

Significance of difference between uptake in normal and tumour-bearing rats: $^{a}P < 0.05$; $^{b}P < 0.01$; $^{c}P < 0.001$.

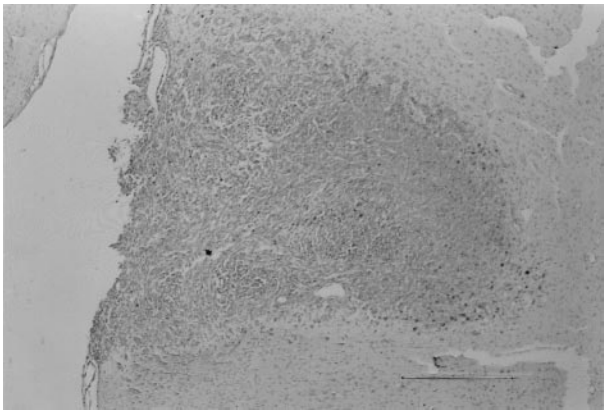

**Figure 1** Typical immunocytochemical staining (brown) of T-cells infiltrating 7-day-old rat brain lesions induced by intracranial implantation of C6 cells square field measuring 0.26 × 0.26 mm (area = 0.068 mm$^2$). Four representative fields, at loci within the centre of the tumour mass, were evaluated per section. Cells which had greater than ten nuclear-associated grains were scored positively. The labelling index for each field was calculated by expressing the number of labelled cells as a percentage of all cells within the field, and the mean labelling index for each animal was determined. For statistical analysis, labelling indices were logarithmically transformed to stabilize the variance in different treatment groups, and compared by analysis of variance with Tukey's method for multiple pairwise comparisons.

### RESULTS

### Pathology

The basic histology of the lesion produced by intracranial implantation of C6 cells was the same in all animals and comprised two types of tissue. The first consisted of interlacing bundles of spindle-shaped cells that had moderate nuclear to cytoplasmic ratios, fairly dense nuclear chromatin with some mitotic figures and rather indistinct cytoplasmic boundaries. The second cell type comprised collections of small mononuclear cells, often perivascular and with the appearance of lymphocytes and macrophages. The C6 cells were negative for glial fibrillary acidic protein (GFAP) and lymphocyte and macrophage markers. The mononuclear population, as expected, marked positively for T-cells and macrophages.

Four days after injection of C6 cells, the size of the lesion was variable and in many cases difficult to distinguish from normal brain. By 7 days, tumours had grown to at least 1.5 mm in diameter and these could be clearly delineated. Larger lesions contained substantial areas of necrosis and in some cases, haemorrhage had occurred at the margins. After 2–3 weeks, some animals showed signs of ataxia. These were immediately killed.

### Inflammatory cell infiltrate

Although the C6 glioma cell line was derived from rat, we observed in our rat model the migration of immune cells to the tumour site. Figure 1 shows the pattern of T-cell-specific staining associated with experimental tumour after 7 days of growth. The distribution of monocyte/macrophage infitrate was similar to that of T-cell distribution – i.e. the greatest concentration of immune cells was at the margins rather than the central portions of the malignant cellular mass. B-cells were absent from the brain sections. After 7 days of tumour growth, T-cells and monocytes/macrophages were evident mainly at the periphery of lesions. In the centres of the tumours, inflammatory cells constituted approximately 6% of the total cellularity. On the basis of these observations, we administered [$^{125}$I]IUdR, directly into brain lesions, 7 days after implantation of C6 cells. The evaluation of tumour and normal brain uptake was performed 24 h after the initiation of the delivery of the radiopharmaceutical.

### Biodistribution

After intratumoural injection of [$^{125}$I]IUdR, no toxicity was observed in any animal. At 24 and 48 h, the retained fraction of

© 2000 Cancer Research Campaign 

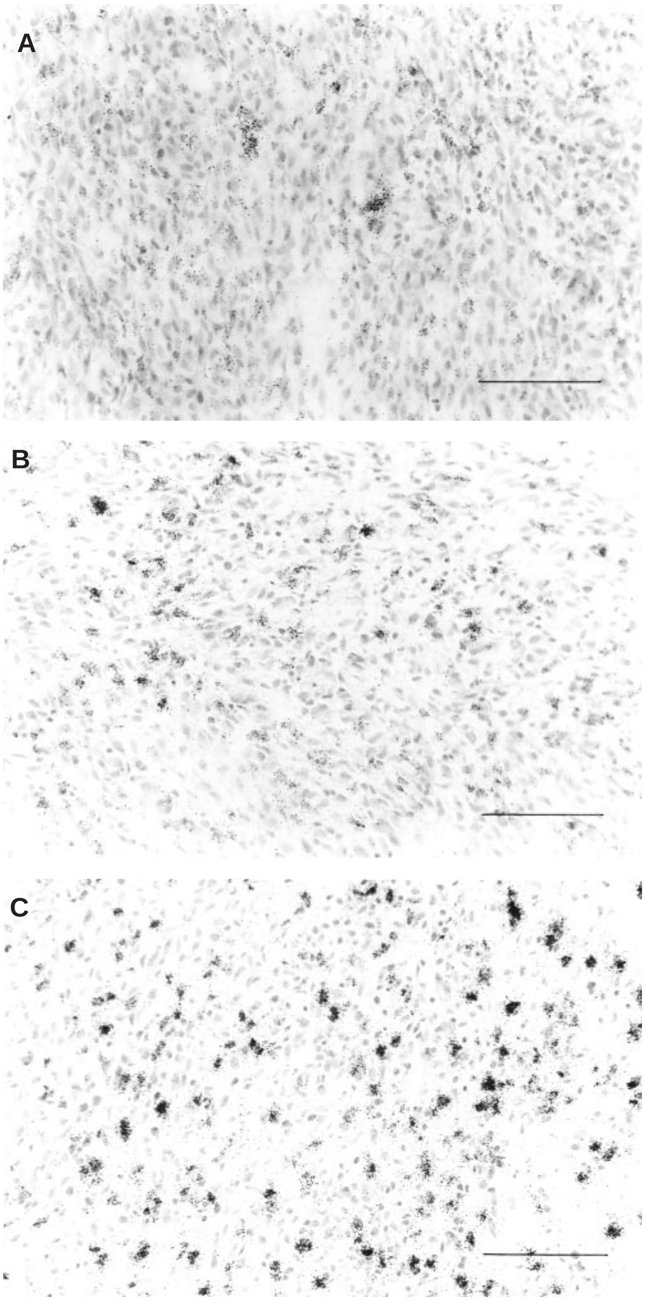

**Figure 2** Autoradiograms of sections of 7-day-old C6 tumours exposed to 0.37 MBq [$^{125}$I]IUdR using alternative delivery methods: (**A**) single intralesional injection; (**B**) slow-release PLGA polymer implant; (**C**) mini-osmotic pump

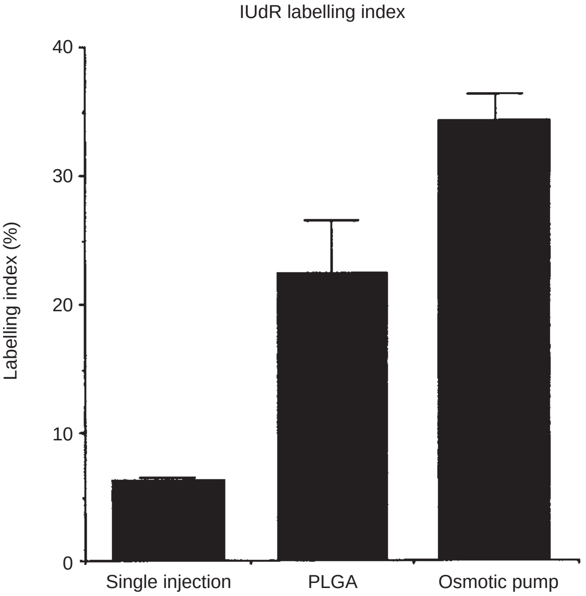

**Figure 3** Labelling index (mean and s.e.m.) of model glioma tumour cells following intracranial delivery of 0.37 MBq of [$^{125}$I]IUdR by single injection ($n$ = 7), by slow release PLGA polymer ($n$ = 3) and by miniosmotic pump ($n$ = 6). The analysis was performed 24 h after the initiation of the delivery of [$^{125}$I]IUdR

injected dose per gram of brain was significantly higher in tumour bearing rats ($P < 0.001$). After 24 h, thyroid, stomach and, to a lesser extent, small intestine were the only normal tissues examined which retained substantial amounts of radioactivity (Table 1). The retention of activity by thyroid was significantly greater in test animals than in control animals at 2 and 24 h ($P < 0.001$ for both time points) after administration of radiopharmaceutical. However, by 48 h, there was no significant difference between thyroid activity in the two groups of rats. It is conceivable that thyroid function could be altered by the presence of an intracranial tumour which could vary the pressure experienced by pituitary or other regulatory ganglia in the brain. This, is turn, could induce variations in thyroid hormone output thereby effecting radiopharmaceutical uptake by thyroid in tumour-bearing rats.

We could detect no activity in DNA isolated from normal tissues of either the test or control group of animals whereas in rats with intracranial tumours, 24 h after injection of radiopharmaceutical, 86.4 ± 2.4% (mean ± s.d.) of the activity associated with brain was DNA-bound. This value increased to 90.1 ± 3.6% (mean ± s.d.) at 48 h. These observations are encouraging from the point of view of therapy. However, the initial rapid loss of IUdR from the brain emphasizes the need for a slow release delivery system.

### IUdR labelling index of experimental tumours

All three delivery systems were well tolerated by the animals; none of the animals exhibited sign of either discomfort or distress. The animals were sacrificed 24 h after the initiation of the administration of [$^{125}$I]IUdR. At this time, the remaining activity associated with PLGA polymer was, in all cases, less than 9% of the loaded dose. The corresponding residual activity in the osmotic pump reservoirs was less than 6% of the loaded dose. From dual labelling pilot studies (autoradiography and immunohistochemistry) it was clear that the immunohistochemical label could not be visualized underneath the silver grains of cells with heavy IUdR uptake. Therefore unequivocal identification of radiolabelled cells expressing leucocyte-specific antigens was not possible. Because the immune cells associated with the intracranial C6 lesions were located mainly at the periphery of the malignant cellular mass, labelling indices were assessed in regions within the body of the tumour.

Autoradiograms of brain sections demonstrated nuclear uptake of the radiopharmaceutical in cells throughout tumour, while normal brain cells remained free from radioactivity. The [$^{125}$I]IUdR labelling indices (% ± s.e.m.) achieved were 6.2 (0.4)

 *© 2000 Cancer Research Campaign*

by single injection, 22.5 (4.1) by slow-release polymer implant (polylactide-glycolide) and 34.3 (2.0) by mini-osmotic pump (Figures 2 and 3). These differences were all statistically significant (pump versus single injection, $P < 0.001$; polymer implant versus single injection, $P < 0.001$; pump versus polymer implant, $P < 0.05$).

### DISCUSSION

Auger electron emitters, such as $^{125}$I, are radiotoxic only to cells which incorporate them into DNA (Kassis et al, 1987). This is because the effective range of Auger electrons is only a few nanometers (Martin and Haseltine, 1981). The thymidine analogue [$^{125}$I]IUdR facilitates the delivery of lethal radiation to dividing cells while sparing quiescent cells. Therefore there is considerable interest in the intralesional delivery of this radiopharmaceutical for the treatment of residual glioma following surgery (Kassis et al, 1996). However, it is recognized that a limitation of short-term administration of [$^{125}$I]IUdR would be sparing of malignant cells not involved in DNA synthesis during the time of exposure to the drug.

In a previous study, using a multicellular tumour spheroid model, we demonstrated that prolongation of incubation time increased cellular accumulation of [$^{125}$I]IUdR (Neshasteh-Riz et al, 1997). This suggested that protracted delivery of the radiopharmaceutical may be used to overcome the difficulty caused by proliferative heterogeneity of gliomas. Brem and co-workers (Brem et al, 1995; Olivi et al, 1996; Sipos et al, 1997) have pioneered the use of sustained release biodegradable polymeric implants containing cytotoxic agents (e.g. carmustine) for the treatment of brain tumours and it has been demonstrated that synthetic implantable, biodegradable polymers hold promise for the controlled release and local delivery of IUdR for radiosensitization of gliomas (Williams et al, 1997). In a recent study involving a rat model of leptomeningeal metastases, continuous infusion of [$^{125}$I]IUdR by means of micro-osmotic pump was found to be more effective than single or intermittent applications of the radiopharmaceutical (Sahu et al, 1997).

The present investigation was designed to compare the extent of cellular uptake of [$^{125}$I]IUdR in experimental tumours achieved by single intralesional injection, slow-release polymer implant and diffusion from osmotic pump. We have demonstrated that significantly greater accumulation of [$^{125}$I]IUdR was achieved in malignant cells by means of sustained delivery than by single intralesional injection and that administration by osmotic pump was superior to release from a biodegradable implant. This confirms our previous findings of enhancement of [$^{125}$I]IUdR uptake in vitro, by prolonged exposure of glioma cell monolayers and spheroids (Neshasteh-Riz et al, 1997) and is in agreement with the previously observed superiority of osmotic pump delivery (Sahu et al, 1997). Recent studies have shown that in vitro there is a rapid release of 40% IUdR in 4 h followed by a slow sustained loss of drug. However, the rate of release of IUdR from PLGA thin films can be finely regulated by varying the thickness of the polymer (Reza and Whateley, 1999). Further refinements to the design of this delivery system are in progress to achieve sustained release of IUdR over a range of time intervals. Extension of the infusion time beyond 24 h to several days should enable greater enhancement of radiopharmaceutical uptake in tumour provided radiolabelled IUdR remains stable. The effectiveness of this approach can be determined using in vivo models (Sahu et al, 1997).

Despite the supposed syngeneity of the tumour cell line employed in our model, an inflammatory infiltrate (T-cells, macrophages and monocytes) was apparent after seven days in regions of the brain adjacent to the site of the malignant lesion. Nonetheless, the density of inflammatory cells in central regions was sufficiently low to allow an evaluation of [$^{125}$I]IUdR uptake into C6 cells. The presence of significant anti-tumour immunity following experimental treatment suggests that the immune system could play a critical role and that the success of novel therapies could be improved by better understanding the part played by the host's immunity. However, a recent study has shown that C6 cells are allogeneic in several strains of rat (Beutler et al, 1999), suggesting that the C6 model may not be useful for experimental therapy.

Twenty-four hours after intratumoural injection of [$^{125}$I]IUdR, 99% of the activity observed after 30 min had cleared from the brains of control animals whereas nuclear uptake occurred in the brains of tumour-bearing animals. After intratumoural injection of [$^{125}$I]IUdR, no toxicity was apparent and the greatest accumulation of the radiopharmaceutical after 48 h occurred in tumour. Activity was evident in the thyroid (indicating incomplete blockade by potassium iodide), in the stomach and small intestine after 2 days. The latter observation is in agreement with pharmacokinetic studies using a rat brain tumour model derived from the rat glioma cell line 9L (Kassis et al, 1990; Kassis and Adelstein, 1996). Encouragingly, concentration of [$^{125}$I]IUdR did not occur in bone marrow.

Heterogeneous proliferative activity of glioma cells is one of the main barriers to the therapeutic use of radiolabelled IUdR. In a previous study of an alternative approach to the eradication of non-cycling malignant cells, we compared the in vitro toxicities of three radioiodoanalogues of IUdR. Ultra-short range Auger electron emitters ($^{125}$I and $^{123}$I) coupled to IUdR were more toxic than [$^{131}$I]IUdR to clonogens derived from single cell cultures and treated in exponential growth phase. In contrast, the long-range β-emitter conjugate [$^{131}$I]IUdR, which provides some 'cross fire' irradiation between cells, was more efficient than the short-range radionuclides in reducing the survival of clonogens derived from multicellular spheroids (Neshasteh-Riz et al, 1998). It was concluded that only cells which were in S phase during the period of incubation with radiopharmaceutical were killed by IUdR conjugated to Auger electron emitters ($^{123}$I and $^{125}$I), whereas [$^{131}$I]IUdR had superior toxicity to clonogenic cells in spheroids due to cross-fire β-irradiation of G0 cells. These findings suggest that a combination of [$^{131}$I]IUdR and [$^{125}$I]IUdR or [$^{123}$I]IUdR might be more effective than [$^{125}$I]IUdR or [$^{123}$I]IUdR alone for the treatment of residual glioma. It is also important to recognize that in the setting of immediate post-surgical resection, regrowth is rapid (Cruickshank, 1997) and a large fraction of cells are actively replicating. In this situation an S phase-specific agent would be valuable, especially if administered by a slow delivery system.

Another means of overcoming the incomplete dose distribution associated with $^{125}$I therapy has been demonstrated using the α-emitter conjugate 5-[$^{211}$At]astato-2′-deoxyuridine ([$^{211}$At]AUdR) (Vaiyanathan et al, 1996). Not only does tumour cell uptake of $^{211}$At enable alpha particle bombardment of neighbouring, untargeted, out-of-cycle cells by cross-fire but each decay of $^{211}$At produces at least ten times the number of DNA double-strand breaks as that obtained per $^{125}$I decay (Walicka et al, 1998). The optimal therapeutic use of radiolabelled deoxyuridine may consist of mixtures of short- and long-range radionuclide derivatives

© 2000 Cancer Research Campaign 

including [$^{125}$I]IUdR, [$^{131}$I]IUdR, [$^{211}$At]AUdR and [$^{123}$I]IUdR. The latter agent, as well as delivering toxic Auger electrons to tumour cells, could, by virtue of its favourable γ-emissions, facilitate imaging to direct external beam irradiation.

In conclusion, we have studied a variety of methods of intracerebral delivery of IUdR to determine which procedure would result in the maximal tumour uptake. Direct injection, implantable osmotic pumps and biodegradable polymers were used to administer [$^{125}$I]IUdR. Autoradiography of brain sections demonstrated nuclear uptake of the radiopharmaceutical in cells throughout tumour while normal brain cells remained free from radioactivity. Labelling was optimal for the pump delivering a given load over 24 h and was substantially better than for a single injection. These results reinforce our in vitro findings and emphasize the need for a protracted delivery system (perhaps extending over several days provided this is within the limits of the stability of [$^{125}$I]IUdR) as a prerequisite for effective treatment. Both direct DNA damage and sensitization to external beam radiotherapy should be increased by prolonged administration of [$^{125}$I]IUdR. Further improvements to the biodegradable implant system are required to ensure adequate, sustained delivery of drug. The systemic biodistribution of labelled iodine after local delivery suggests that this approach to treatment is likely to carry minimal toxicity. These findings are extremely encouraging for the development of a sustained release system for radiolabelled IUdR for use in the treatment of intracranial tumours particularly in the immediate post-operative setting.

### ACKNOWLEDGEMENTS

This work was supported by the Cancer Research Campaign (Grant No. SP1866/0302) and by the European Community (BIOMED Contract BMH4-CT98-3297).

### REFERENCES

Baranowska-Kortylewicz J, Makrigiorgos GM, Van Den Abbeele AD, Berman RM, Adelstein SJ and Kassis AI (1991) 5[$^{123}$I]iodo-2′-deoxyuridine in the radiotherapy of an early ascites tumor model. *Int J Radiat Oncol Biol Phys* **21**: 1541–1551

Beutler AS, Banck MS, Wedekind D and Hedrich HJ (1999) Tumor gene therapy made easy: allogeneic major histocompatibility complex in the C6 rat glioma model. *Human Gene Ther* **10**: 95–101

Brem H, Piantadosi S, Burger PC, Walker M, Selker R, Vick NA, Black K, Sisti M, Brem S, Mohr G, Muller P, Morawetz R and Schold SC (1995) Placebo-controlled trial of safety and efficacy of intraoperative controlled delivery by biodegradable polymers of chemotherapy for recurrent gliomas. *Lancet* **345**: 1008–1012

Cruickshank GS (1997) The use of SPECT in the analysis of brain tumours. In: *SPECT Imaging of the Brain*, Duncan R (ed), pp. 161–178. Kluwer Academic: Dodrecht

Cunningham SH, Mairs RJ, Wheldon TE, Welsh PC, Vaidyanathan G and Zalutsky MR (1998) Radiotoxicity to neuroblastoma cells and spheroids of beta-, alpha- and Auger electron-emitting conjugates of benzylguanidine. *Br J Cancer* **77**: 2061–2068

Kassis AI, Fayad F, Kinsey BM, Sastry KSR, Taube RA and Adelstein SJ (1987) Radiotoxicity of $^{125}$I in mammalian cells. *Radiat Res* **111**: 305–318

Kassis AI, Wen PW, Van den Abbeele AD, Baranowska-Kortylewicz J, Makrigiorgos GM, Metz KR, Matalka KZ, Cook CU, Sahu SK, Black PM and Adelstein SJ (1998) 5-[$^{125}$I]iodo-2′-deoxyuridine in the radiotherapy of brain tumors in rats. *J Nucl Med* **39**: 1148–1154

Kassis AI and Adelstein SJ (1996) 5-[$^{125}$I]iodo-2′-deoxyuridine in the radiotherapy of solid CNS tumors in rats. *Acta Oncol* **35**: 935–939

Kassis AI, Tumeh SS, Wen PYC, Baranowska-Kortylewicz J, Van Den Abbeele AD, Zimmerman RE, Carvalho PA, Garada BM, Desisto WC, Bailey NO, Castronovo FP, Mariani G, Black PM and Adelstein SJ (1996) Intratumoral administration of 5[$^{123}$I]iodo-2′-deoxyuridine in a patient with a brain tumor. *J Nucl Med* **37**: 19S–22S

Klecker RW Jr, Jenkins JF, Kinsella TJ, Fine RL, Strong JM and Collins JM (1985) Clinical pharmacology of 5-iodo-2′-deoxyuridine and 5-iodo-uracil and endogenous pyrimidine modulation. *Clin Pharmacol Ther* **38**: 45–51

Larsen RH, Vaidyanathan G and Zalutsky MR (1997) Cytotoxicity of α-particle-emitter 5-[$^{211}$At]astato-2′-deoxyuridine in human cancer cells. *Int J Radiat Biol* **72**: 79–90

Martin RF and Haseltine WA (1981) Range of radiochemical damage to DNA with decay of iodine-125. *Science* **213**: 896–898

Menei P, Boisdron-Celle M, Croue A, Guy G, Benoit JP (1996) Effect of stereotaxic implantation of biodegradable 5-fluorouracil-loaded microspheres in healthy and C6 glioma-bearing rats. *Neurosurgery* **39**: 117–123

Neshasteh-Riz A, Angerson WJ, Reeves JR, Smith G, Rampling R and Mairs RJ (1997) Incorporation of iododeoxyuridine in multicellular glioma spheroids: implications for DNA-targeted radiotherapy using Auger electron emitters. *Br J Cancer* **75**: 493–499

Neshasteh-Riz A, Mairs RJ, Angerson WJ, Stanton PD, Reeves JR, Rampling R, Owens J and Wheldon TE (1998) Differential cytotoxicity of [$^{123}$I]IUdR, [$^{125}$I]IUdR and [$^{131}$I]IUdR to human glioma cells in monolayer or spheroid culture: effect of proliferative heterogeneity and radiation cross-fire. *Br J Cancer* **77**: 385–390

O'Donoghue JA and Wheldon TE (1996) Targeted radiotherapy using Auger electron emitters. *Phys Med Biol* **41**: 1973–1979

Olivi A, Ewend MG, Utsuki T, Tyler B, Domb AJ, Brat DJ and Brem H (1996) Interstitial delivery of carboplatin via biodegradable polymers is effective against experimental glioma in the rat. *Cancer Chem Pharmacol* **39**: 90–96

Reza MS and Whateley TL (1998) Iodo-2′-deoxyuridine (IUdR) and $^{125}$IUdR loaded biodegradable microspheres for controlled delivery to the brain. *J Microencapsulation* **15**: 789–801

Reza S and Whateley TL (1999) Biodegradable thin films for sustained delivery to the brain. *Drug Delivery* (in press)

Sahu SK, Wen PYC, Foulon CF, Nagel JS, Black PMcL, Adelstein SJ and Kassis AI (1997) Intrathecal 5-[$^{125}$I]iodo-2′-deoxyuridine in a rat model of leptomeningeal metastases. *J Nucl Med* **38**: 386–390

Sipos EP, Tyler B, Piantadosi S, Burger PC and Brem H (1997) Optimizing interstitial delivery of BCNU from controlled release polymers for the treatment of brain tumors. *Cancer Chem Pharmacol* **39**: 383–389

Vaidyanathan G, Larsen RH and Zalutsky MR (1996) (5-[$^{211}$At]astato-2′-deoxyuridine, an α-particle-emitting endoradiotherapeutic agent undergoing DNA incorporation. *Cancer Res* **56**: 1204–1209

Vertosick FT, Selker RG, Grossman SJ and Joyse JM (1994) Correlation of thallium-201 single photon emission computed tomography and survival after treatment failure in patients with malignant glioma. *Neurosurgery* **34**: 396–401

Walicka MA, Vaidyanathan G, Zalutsky MR, Adelstein SJ and Kassis AI (1998) Survival and DNA damage in chinese hamster V79 cells exposed to alpha particles emitted by DNA-incorporated astatine-211. *Radiat Res* **150**: 263–268

Whateley TL, Rampling R, Robertson L, Crossan IM, Fallon PA, Plumb JA and Kerr DJ (1995) Biodegradable systems for sustained delivery of drugs to brain-tumors. *J Cell Biochem* **S19A**: p. 178.

Williams JA, Dillehay LE, Tabassi K, Sipos E, Fahlman C and Brem H (1997) Implantable biodegradable polymers for IUdR radiosensitisation of experimental human malignant glioma. *J Neurooncol* **32**: 181–192

Zhu J, Zhang L, Hanisch UK, Felgner PL and Reszka RA (1996) Continuous intracerebral gene delivery system for in vivo liposome-mediated gene therapy. *Gene Ther* **3**: 472–476

© 2000 Cancer Research Campaign
